# Impact of Sports Education Model in Physical Education on Students’ Motivation: A Systematic Review

**DOI:** 10.3390/children8070588

**Published:** 2021-07-11

**Authors:** Ricardo Tendinha, Madalena D. Alves, Tiago Freitas, Gonçalo Appleton, Leonor Gonçalves, Andreas Ihle, Élvio R. Gouveia, Adilson Marques

**Affiliations:** 1 Faculty of Human Kinetics, University of Lisbon, 1499-002 Cruz Quebrada, Portugal; ricardo.martins1@edu.ulisboa.pt (R.T.); madalenadalves@hotmail.com (M.D.A.); tiago.freitas2@campus.ul.pt (T.F.); poiaresappleton@hotmail.com (G.A.); leonorgoncalves1803@hotmail.com (L.G.); 2 Center for the Interdisciplinary Study of Gerontology and Vulnerability, University of Geneva, 1205 Geneva, Switzerland; Andreas.Ihle@unige.ch; 3 Swiss National Centre of Competence in Research LIVES—Overcoming Vulnerability: Life Course Perspectives, 1015 Lausanne, Switzerland; 4 Department of Psychology, University of Geneva, 1205 Geneva, Switzerland; 5 Department of Physical Education and Sport, University of Madeira, 9000-390 Funchal, Portugal; erubiog@staff.uma.pt; 6 Interactive Technologies Institute, LARSyS, 9020-105 Funchal, Portugal; 7 CIPER, Faculty of Human Kinetics, University of Lisbon, 1499-002 Cruz Quebrada, Portugal; 8 ISAMB, University of Lisbon, 1649-004 Lisbon, Portugal

**Keywords:** SEM, sports education, motivation, student behavior, attitude, self-determination-theory

## Abstract

Background: Research has suggested that applying the Sport Education Model (SEM) in Physical Education (PE) increases students’ motivation. However, it is important to systematize this evidence to have a clearer idea. Therefore, this study aimed to analyze the impact of the SEM on the students’ motivation. Methods: A systematic review with a narrative synthesis was performed. In March 2021, an articles search was conducted in PubMed, Scopus, and Web of Science. Eligibility criteria were: longitudinal or experimental study design; outcomes included PE settings; results reported the relationship between the SEM and students’ motivation. Results: Fourteen studies were included, totaling 2146 students. The majority of the studies indicated a significant association between the SEM and motivation, particularly in autonomy and more enjoyment toward PE. Conclusions: This review supports that the SEM has a positive impact on motivation. The SEM offers a wide range of opportunities for students to develop more self-determined motivated behavior in PE classes. Therefore, the SEM should be considered when developing or adapting existing PE programs to promote students’ intrinsic motivation to engage in physical activity.

## 1. Introduction

The Sport Education Model (SEM) offers the nearest approach to sports experience adapted to the school context [1]. The model was created because physical education (PE) classes should not be limited to teaching techniques and tactics from multiple sports. PE should make students cultivate their habits of exercising and improve their sports culture along the way [1]. The SEM is a curriculum and instructional model created to provide richer sports-related experiences for students during PE classes [2]. The model is organized around a series of characteristics, which are, (1) units are considered seasons, (2) students are members of intact teams, (3) participation in formal competition, (4) students maintain roles beyond players, (5) formal records are kept, and (6) students participate in a culminating event [2].

Motivation is important to influence students’ learning [3]. Especially, intrinsic motivation has a positive impact on students’ behavior and learning during PE [4]. Some studies related to intrinsic motivation in PE and sports have indicated that this construct is positively associated with self-effort and predisposition to participate in future physical activities [5,6]. For the teacher to improve these capacities in the students, they may impose tasks related to personal control or self-competence that will improve several adaptive responses to motivational imposes [7].

Most research acknowledges that the SEM as a more effective model than the traditional and direct instruction model in various factors like students’ attitudes, motivation, or self-determination towards PE [8], mainly in low-performing students [9]. According to the self-determination theory, intrinsic motivation is promoted by fulfilling competence, autonomy, and relatedness [10]. The SEM principles are related to these concepts, which may explain why investigations have purposed it to be related to greater students’ motivation than traditional PE models. For example, characteristics of the SEM, such as the festive finale, the student-centered approach and autonomy, engagement, and peer relationships in PE, can contribute to greater motivation [11,12]. In addition, the use of dynamic roles during the classes is viewed as an aspect with a very high relation to students’ motivation [13]. Contrary, some research has proposed that the SEM focus on formal competition has a detrimental effect on students’ motivation [14].

One of the objectives of PE is the increase of physical activity (PA) levels and motivation for PA in and out of school. Research suggests that students’ motivation in PE following the SEM is significantly higher than students receiving traditional PE [8,9,15,16]. However, to better ascertain the role of the SEM on motivation, it is important to summarize the existing evidence. Thus, the objective of this study was to analyze the impact of the SEM on the motivation for PA.

## 2. Materials and Methods

### 2.1. Search Strategy and Inclusion Criteria

**Figure 1:**
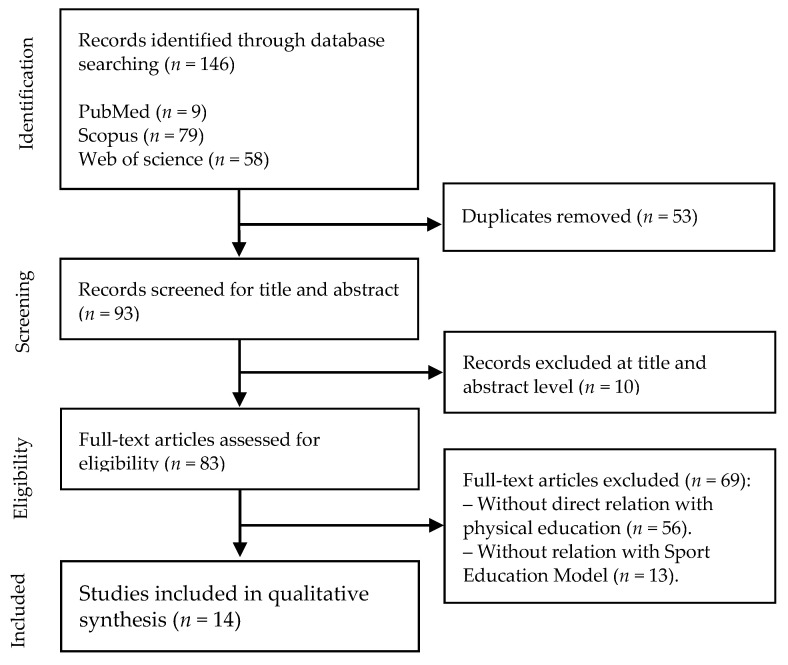
Flow diagram of study selection.

The review was performed following the Preferred Reporting Items for Systematic Reviews and Meta-Analyses (PRISMA) guidelines [17] and is presented in Figure 1. Articles that studied the relationship between the SEM in PE classes and students’ motivation served as a basis for this review [8,9,18].

The inclusion criteria were: (a) prospective and experimental study design (study design criterion); (b) effect of the SEM on students’ motivation during PE classes (relationship criterion); (c) PE students (participants’ criterion); (d) articles published in English, Portuguese, or Spanish (language criterion); (e) articles were left out if they did not meet inclusion criteria or did not have findings associated to the inclusion criteria (exclusion criteria).

In March 2021, a search was made in PubMed, the Web of Science, and Scopus. The search was performed in the two databases using the terms “SEM” OR “sport education model” OR “sport education” AND “motivation” OR “student behaviour” OR “attitude” OR “interest” OR “predisposition” OR “self-determination-theory”. Two reviewers screened titles and abstracts to identify articles that met the inclusion criteria. Five authors read the articles and decided whether they should be included in the analysis or excluded. The studies inclusion decision was consensual. In cases of disagreement, the decision was made by consent.

### 2.2. Data Extraction

The authors’ name, year of publication, study design, participant characteristics, country, methods of SEM evaluation, instruments to assess motivation, and main findings were extracted from each article. Five authors carried out the extraction, and another author verified coding.

### 2.3. Synthesis of Results

The review analyzed the relationship between the SEM and students’ motivation. Among the studies, the different parameters analyzed were homogeneous. The study details, including design, measures, sample size, participant characteristics, and results, are presented consistently.

## 3. Results

### 3.1. Overview of Articles and Study Background

The majority of the 14 studies were performed in Spain (9), China (3), United States (1), and England (1). All, except one study [19], used a theoretical framework of motivation in their investigation. All 14 studies included descriptions of the SEM, discuss the relationship between the SEM and motivation and enjoyment in PE (8), social affiliation (2), PA participation (2), and some other motivational outcomes (7). The greatest number of articles achieved this assessment criterion.

### 3.2. Participants and Setting

The total student sample was 2146 (n = 1132 boys, n = 950 girls) from the 14 included studies. One study did not specify its sample about sex [19]. Most studies were performed in high schools (aged 14–17) [14,15,16,18,19,20,21,22,23]. While most studies examined the SEM in a co-educational context, one examined boys in a single-sex PE context [14]. Moreover, three studies described the ethnicity of the participants [14,23,24].

Six studies did not describe participants’ eligibility criteria and selection [13,18,19,21,22,25]. Twelve of them included information about teachers’ experiences in sport education and/or PE [8,13,14,15,18,19,20,21,22,23,24,25], but only seven reported students’ experiences [9,14,15,16,20,21,25].

All studies but two [13,22] included more than one class regarding PE settings. Three studies did not specify how many classes are included [15,16,20]. Eight studies included one school, two included two schools [23,25], while four look at the setting of secondary educational centers, instead of regular schools [15,18,20,22].

### 3.3. Program Design and Implementation

Seven studies used a quasi-experimental design [8,9,13,18,20,22,25] and three nonequivalent control-group designs [14,19,23] to investigate the motivational impact of a the SEM program by including one intervention and one control group, except for one study that had three different intervention groups [8]. Two studies used a pre-experimental pre-/post-test design [15,16], one study used a crossover design [21], and another one used a cluster randomized study design [24].

Ten of the studies included one sport [8,9,13,14,16,18,19,20,22,25], and the other four included two to six sports in their program. Concerning the program duration, 11 studies examined one season [8,9,13,14,15,16,18,19,20,22,25]; Gil-Arias, Harvey, Cárceles, Práxedes, and Del Villar [21] investigated two seasons; Wallhead, Garn, and Vidoni [23] investigated four seasons; and Choi, Sum, Leung, Wallhead, Morgan, Milton, Ha, and Sit [24] investigated seven seasons. The season length ranged from 8 to 25 lessons, lasting from 5 to 16 weeks. The lesson frequency ranged from one to three lessons per week. The lessons were mostly 40–60 min long, although some programs used a double-lesson format of 90 min [24].

The sport education programs were frequently delivered by one to three teachers with teaching experience. The majority of teachers had more than five years of teaching experience. However, only less than half of them had prior teaching experience in sports education.

### 3.4. Main Results

Thirteen studies (93%) reported a significant relationship between the SEM and students’ motivation [8,9,13,14,15,16,19,20,21,22,23,24,25] (Table 1). Higher autonomy and more enjoyment toward PE sessions were associated with the SEM. Furthermore, the SEM promoted an inclusive PE learning environment [16,25] and showed that students became more interested than direct instruction or the traditional education model [19,21]. However, in one study, the students’ motivation to practice sports was not affected by the SEM [18]. Table 1 offers an overview of the 14 studies included in the review.

## 4. Discussion

This systematic review aimed to analyse the SEM on students’ motivation. The results of 13 articles included in this study reveal a significant relationship between the SEM and students’ motivation, reporting greater autonomy and more enjoyment during PE sessions and an inclusive PE learning environment. Only one article states that motivation for sports practice is not affected by the SEM.

This research collectively offers practical implications for both students and teachers during their PE classes. Starting with the students, we found that the implementation of the SEM facilitates the autonomy-supportive instructional context during classes [26]. The students show a significant decrease in their perception of a disempowering motivational climate in their classes and improving their PA levels [24]. Therefore, the SEM is seen as a model which improves students’ motivational needs [8,9,15]. the SEM is considered efficient in developing students’ self-determination motivation [14,24,26]. This motivation is related to more autonomy satisfaction and a better learning experience [27,28,29,30]. Additionally, the SEM can expose students to an opportunity to work as a team. Research indicates that teamwork is a key factor for improving motivation to learning [31], giving students an opportunity to develop their sense of responsibility [19].

It is important to observe that the teachers’ implementation of the model is a game-changer in the success of the intervention. The primary goal of the SEM is to develop competent sportspersons [1]. Still, the level of compliance with the model’s critical factors to establish an authentic teaching-learning context requires that teachers follow a well-defined intervention protocol. This directly impacts students’ success, especially in skill and tactical performances [14]. Concerning the motivational component, teachers may use different strategies like giving additional points during the season to reinforce the importance of fair play [8]. Importantly, promoting teams for a whole season, which can provide positive and constructive feedback to their colleagues, is central to ensure that the principles of the fair-play are established [28].

This study reinforces the idea that the SEM can significantly improve students’ motivation and engagement throughout PE classes. Therefore, the SEM should be considered when developing or adapting existing PE programs in order to promote intrinsic motivation, and possibly present and future engagement in PA. For that to happen, teachers must adapt their approach to PE by following key critical features to the SEM development in schools. Teachers give students a deeper approach to each sport, and that fact makes them improve and know better all situations that occur in that specific sport. Another point is the competition and the teams being structured at the beginning of the year, which makes them improve, not only by themselves but by improving the team as a group, to have better results [2].

Summarizing, this review supports that the SEM positively impacts motivation, which keeps the students’ predisposition to be active and mentally ready to improve at each PE class. Thus, the SEM seems to enhance the current PA and promote some sports habits for their future. Dynamic roles used throughout PE classes make the students more motivated because they can perform a role where they are good and give effective help to their team [13]. These findings are of importance and reflect the potential of the SEM.

Some limitations have to be to be acknowledged for future research. First, the sample size in most of the studies analyzed was considered small. As a result, it would be necessary to include many students from different schools in future studies. By increasing the sample, more significant differences may emerge due to the improved statistical power. Moreover, most studies investigated team sports in SE programs. Only three studies used individuals’ sports to apply the pretend methodology. Therefore, more research is necessary to study whether individual sports in the SEM leads to different results from team sports. Most studies included teachers with no previous experience with the SEM. Consequently, differences in the study design may serve as a confounding factor because the motivational styles of different teachers can cause differences in students’ motivational outcomes. The extent to which the teacher employs autonomous and controlling behaviors could be studied to offer a potentially richer picture of why some groups may not demonstrate similar changes in motivation to others. Therefore, information about the teacher-created motivational climate should be looked at for potential differences in the motivational impacts of SE taught by different teachers. Regarding data collection, the effects of the intervention were evaluated through self-reported questionnaires. Hence, future studies should use other measurement methods, such as qualitative and observational methodology. Moreover, collecting and analyzing objective data such as motor skills, physical fitness, and PA could provide more evidence on the actual behavioral changes resulting from the impacts of SE. In addition, the application of teaching models was applied during six to eight weeks in most of the studies, one season. It would be valuable to conduct longitudinal studies to investigate students’ motivation over a longer time frame. Longer periods should be used in future studies, especially for post-assessment after an intervention. More follow-up data can show long-term changes in motivational outcomes beyond the instant effects of SE.

## 5. Conclusions

This review shows that the SEM offers a large range of opportunities for students to develop more self-determined motivated behaviour in PE class with high levels of responsibility and engagement. In this aspect, the SEM can be considered a useful methodological instrument to change the current trend of declines in motivation and participation in PA by adequately implementing the SEM in PE classes.

## Figures and Tables

**Table 1:** Study characteristics and main findings.

Source	Country	Study Design, Sample Characteristics (n, Sex, Age), Recruitment	Sport Education Model Experience	Data Assessment (Instruments to Assess Motivation)	Main Findings
Burgueño, Medina-Casaubón, Morales-Ortiz, Cueto-Martín and Sánchez-Gallardo [20]	Spain	Quasi-experimental study. Pre-and post-test measures and intra- and inter-group analysis. 44 high school students (22 boys, M_age_ = 16.32 ± 0.57).	Following the structural guidelines of the SEM established by Siedentop et al. (2011) for 12 sessions of basketball teaching.	Situational Motivation Scale.	The SEM significantly improved the level of intrinsic motivation and identified regulation about TEM. SE has significantly reduced external regulation and amotivation in students regarding TTM.
Burgueño, Cueto-Martín, Morales-Ortiz and Medina-Casaubón [15]	Spain	Pre-experimental pre-/post-test design. 75 high school students (38 boys, M_age_ = 16.75 ± 0.87).	The intervention programme under SE conditions included 3 classes, twelve 50-min lessons each, twice per week in regular PE schedule. Based on the preference of the three PE teachers, indoor soccer, volleyball and basketball were taught.	Perceived Locus of Causality Scale. Achievement Goal Questionnaire-Physical Education (Spanish version). Social Goal Scale Physical Education (Spanish version).	The SEM was a pedagogical model that favours the adequate motivational response of high school students in terms of self-determination, motivational achievement, and social motivation in the sports teaching-learning process in PE classes.
Chenchen, Rong and Shuaijing [19]	China	Study with nonequivalent pre-test–post-test. Two groups: IG (the SEM group) n = 36 and CG (traditional sport Model) n = 28; Aged 16–17 years old from a senior high school in China.	Students participated in one lesson per week for 16 weeks in a semester, and each lesson should last for 40 min of table tennis classes taught following the SEM.	Questionnaire of student’s attitude and interviews.	The learning attitudes of students in SE class including cognitive, emotional, and behaviour disposition improved significantly after the season.
Choi, Sum, Leung, Wallhead, Morgan, Milton, Ha and Sit [24]	China, Hong Kong	A Cluster-randomised study design. 372 participants. Two groups: IG (the SEM group) n = 188 and CG n = 184. M_age_ 18.5 years. 95% of the study sample were Chinese. 70% of the study sample were male.	SE seasons were designed for badminton, basketball, football handball, physical conditioning, swimming, and volleyball. Each SE season included ten 90-min lessons, 1-day per week.	Situational Motivation Scale (SIMS). Physical activity enjoyment scale (PACES). Empowering and disempowering motivational climate questionnaire in PE (EDMCQ—PE).	The SE group presented higher scores in the internalised regulations of intrinsic and identified regulation motivations. They also had lower scores in external and amotive regulations.
Cuevas, García-López and Serra-Olivares [18]	Spain	Quasi-experimental design. Two groups: IG (the SEM group) n = 43; and CG (traditional PE lessons) n = 43. 86 PE students (49 girls) between 15 and 17 years of age (M_age_ = 15.65; SD = 0.78).	The teaching unit on volleyball consisted of 19 55-min sessions (two/week in the regular PE schedule) structured based on the SEM.	Questionnaire for Evaluating Motivation in Physical Education (CMEF). Sport Satisfaction Instrument (SSI). Intention to be Physically Active Scale (IPAS).	The results showed improvements in intrinsic motivation in the SEM intervention group.
García-González, Abós, Diloy-Peña, Gil-Arias and Sevil-Serrano [16]	Spain	Pre-experimental pre-/post-test design. A final sample of 49 students, M_age_ = 15.5 ± 0.57, 49% female from secondary education level.	A hybrid SE/TGfU volleyball teaching unit was applied twice per week over five weeks (10 lessons of 50 min).	The Basic Psychological Needs Support Questionnaire in PE. Basic Psychological Need in Exercise Scale (BPNES). Novelty Need Satisfaction Scale (NNSS). Perceived Variety in Exercise questionnaire (PVE).	This hybrid SE/TGfU could improve the students’ motivation during the PE classes, particularly those who showed an early low or moderate self-determined motivation at the beginning of the intervention. This model could bring more positive experiences to the students and be more inclusive.
Gil-Arias, Harvey, Cárceles, Práxedes and Del Villar [21]	Spain	A crossover design was utilised, using the technique of counterbalancing. 55 students (M_age_ = 15.45 ± 0.41, min. 27 females from Secondary Education school.	The intervention was conducted over eight weeks (two months) for a total of 16 lessons and focused on the team sports of volleyball and ultimate frisbee. One group experienced a hybrid SE/TGfU unit, followed by a unit of direct instruction. A second group had the units in the opposite order.	Perceived Locus of Causality. Basic Psychological Needs in Exercise Scale. Enjoyment in Sport Scale. The intention to be physically active scale was administered to participants.	A hybrid model of TGfU/SE stimulated increases in autonomy, relatedness, competence, autonomous motivation, enjoyment, and intention to be more active compared to direct instruction.
Gil-Arias, Harvey, García-Herreros, González-Víllora, Práxedes and Moreno [25]	Spain	Pre-intervention/post-intervention quasi-experimental design. IG (the SEM group) n = 148; M_age_ = 10.39 ± 0.48, 71 females; G (direct instruction) n = 144; M_age_ = 10.43 ± 0.49, 69 females. Students were in their fifth year of elementary school	A hybrid SE/TGfU basketball teaching unit was conducted during 16 lessons (8 weeks), 50 min twice a week.	Perceived Locus of Causality Questionnaire. PA Class Satisfaction Questionnaire. Autonomy-Supportive Coaching Strategies Questionnaire. BPNs in exercise scale. Relationship goals questionnaire-friendship version.	A hybrid TGfU/SE unit encouraged an autonomy-supportive, inclusive, and equitable PE learning environment. All students have chances to increase their commitment, enjoyment, and social interactions within PE lessons.
Kao and Luo [9]	Taiwan, China	Quasi-experimental design. IG (the SEM group) n = 59; M_age_ = 21.42 ± 0.75, 32 men; CG (direct instruction) n = 56; M_age_ = 21.38 ± 0.73, 29 men.	A SE-based badminton teaching unit was conducted over 10 weeks.	Elective Motivation Scale of Physical Education Curriculum (EMSPEC).	Students’ elective motivation toward PE improved and was higher than those who received direct instruction. The SEM also increased the elective motivation of low-performing students.
Medina-Casaubón and Burgueño [22]	Spain	Quasi-experimental design. 44 students (22 girls, M_age_ 16.32 ± 0.57). IG (the SEM group) n = 22; CG (traditional teaching group) n = 22.	A SE basketball teaching unit was conducted for 12 lessons of 55 min.	The Questionnaire to Support Basic Psychological Needs in Physical Education.	the SEM improved the perceived level of autonomy support and structure in the inter-group analysis and the intra-group.
Méndez-Giménez, Fernández-Río and Méndez-Alonso [8]	Spain	Quasi-experimental design with three levels of treatment: (1) Traditional model = 110, (3) SE- with conventional resources: N = 107, and (3) SE- with self-made materials: N = 78. In total 295 students (159 males), M_age_ = 14.2 ± 1.68. Participants belonged to different class groups from 7th to 11th grade	A SE Ultimate-Frisbee learning unit of 12 sessions of 50 min each. Two SE approaches were considered: (1) SE with conventional resources and (2) SE with self-made materials.	Achievement Goal Questionnaire-Physical Education (AGQ-PE). Friendship Goals in Physical Education Questionnaire. Basic Psychological Needs in Exercise Scale (BPNES).	Both the SEM groups reported improvements over time in autonomy, competence, and relatedness to others, versus the only improvement in autonomy in the traditional model. The SEM was shown to be more effective than the traditional method at the motivational and attitudinal levels.
Puente-Maxera, Méndez-Giménez and de Oieda [13]	Spain	Quasi-experimental, pre-test, and post-test measures study. 36 students (17 women; mean age 11.36 ± 0.59). Participants belonged to elementary school	A SE handball teaching unit of 10 sessions of 60 min each.	Motivation Orientation (GOES). Climate Motivation (CMI). Basic Psychological needs satisfaction (EMMD). Interviews.	the SEM produced an oriented climate to the task. Dynamic roles were shown as a powerful methodological strategy for students’ motivation.
Wallhead and Ntoumanis [14]	England	Non-equivalent control group (IG (the SEM) n = 25; CG= (traditional teaching approach), n = 26. In total, 51 boys (46 Caucasians and 5 Asian descent; M_age_ 14.3 ± 0.48).	8-week intervention of SE basketball teaching unit.	Enjoyment, Effort, and Perceived Competence (IMI). Achievement goal orientations (TEOSQ). Perceived Autonomy (ASRQ). Perceptions of motivational climate (LAPOPECQ).	Students in the SEM had significantly higher post-intervention enjoyment and perceived effort than those taught with the traditional PE approach.
Wallhead, Garn and Vidoni [23]	United States, Midwestern	Non-equivalent control-group design (IG = the SEM approach; CG = multi-activity model of instruction). 568 students from 2 high schools (310 girls; M_age_ 14.75 ± 0.48; ethnic minority students 20%-30%).	Four 25-lesson seasons of floor hockey, volleyball, team handball, and basketball were taught using the SE approach.	Perceived Locus of Causality Questionnaire (PLCQ). Academic Motivation Scale (AMS). Intrinsic Motivation Inventory (IMI).	The SEM group reported greater increases in perceived effort and enjoyment of the program than the students taught within the multi-activity model. Those positive affective outcomes were enabled by the development of more autonomous forms of motivation.

Abbreviation: ACQ-PE, Achievement Goal Questionnaire-Physical Education; AMS, Academic Motivation Scale; ASRQ, Academic Self-Regulation Questionnaire; BPNES, Basic Psychological Needs in Exercise Scale; CBAS, Coach Behavior Assessment System; CG, control group; CMI, Escala de Percépcion del Clima Motivacional de los Iguales; EMMD, Escala de los Mediadores Motivaciones en el Deporte; EMPSEC, Elective Motivation Scale of Physical Education Curriculum; GOES, Escala de las Orientaciones de Meta en el Ejercicio; IMI, Intrinsic Motivation Inventory; LAPOPECQ, Learning and Performance Orientations in Physical Educations Classes Questionnaire; M_age_, mean age; PA, physical activity; PE, Physical Education; SD, standard deviation; SE, sport education; the SEM, Sport Education Model; TEM, Traditional Education Model; TEOSQ—Task and Ego Orientation in Sport Questionnaire; TGfU, Teaching Games for Understanding; TSM, Traditional Sports Model.

## References

[1] Siedentop D. (1994). Sport Education: Quality PE through Positive Sport Experiences.

[2] Siedentop D., Hastie P., Van der Mars H. (2011). Complete Guide to Sport Education.

[3] Chen A. (2001). A Theoretical Conceptualization for Motivation Research in Physical Education: An Integrated Perspective. Quest.

[4] Mitchell S. (1996). Relationships between Perceived Learning Environment and Intrinsic Motivation in Middle School Physical Education. J. Teach. Phys. Educ..

[5] Ntoumanis N. (2001). A self-determination approach to the understanding of motivation in physical education. Br. J. Educ. Psychol..

[6] Pelletier L., Tuson K., Fortier M., Vallerand R., Briére N., Blais M. (1995). Toward a New Measure of Intrinsic Motivation, Extrinsic Motivation, and Amotivation in Sports: The Sport Motivation Scale (SMS). J. Sport Exerc. Psychol..

[7] Miller K., Deci E., Ryan R. (1988). Intrinsic Motivation and Self-Determination in Human Behavior. Contemp. Sociol..

[8] Méndez-Giménez A., Fernández-Río J., Méndez-Alonso D. (2015). Sport education model versus traditional model: Effects on motivation and sportsmanship. Rev. Int. Med. Cienc. Act. Fis. Deporte.

[9] Kao C.C., Luo Y.J. (2019). The influence of low-performing students’ motivation on selecting courses from the perspective of the sport education model. Phys. Educ. Stud..

[10] Ryan R., Deci E. (2017). Self-Determination Theory: Basic Psychological Needs in Motivation, Development and Wellness.

[11] Perlman D. (2010). Change in Affect and Needs Satisfaction for Amotivated Students within the Sport Education Model. J. Teach. Phys. Educa..

[12] Hassandra M., Goudas M., Chroni S. (2003). Examining factors associated with intrinsic motivation in physical education: A qualitative approach. Psychol. Sport Exerc..

[13] Puente-Maxera F., Méndez-Giménez A., de Oieda D.M. (2018). Sport education model and roles’ dynamics. Effects of an intervention on motivational variables of elementary schools’ students. Cult. Cienc. Deporte.

[14] Wallhead T.L., Ntoumanis N. (2004). Effects of a sport education intervention on students’ motivational responses in physical education. J. Teach. Phys. Educ..

[15] Burgueño R., Cueto-Martín B., Morales-Ortiz E., Medina-Casaubón J. (2020). Influence of sport education on high school students’ motivational response: A gender perspective. Retos.

[16] García-González L., Abós Á., Diloy-Peña S., Gil-Arias A., Sevil-Serrano J. (2020). Can a hybrid sport education/teaching games for understanding volleyball unit be more effective in less motivated students? An examination into a set of motivation-related variables. Sustainability.

[17] Moher D., Liberati A., Tetzlaff J., Altman D., Group T.P. (2009). Preferred Reporting Items for Systematic Reviews and Meta-Analyses: The PRISMA Statement. PLoS Med..

[18] Cuevas R., García-López L.M., Serra-Olivares J. (2016). Sport education model and self-determination theory: An intervention in secondary school children. Kinesiology.

[19] Xu C., Gao R., Xu S. (2019). Impact of a sport education season on students’ table tennis skills and attitudes in China’s high school. Int. J. Inf. Educ. Technol..

[20] Burgueño R., Medina-Casaubón J., Morales-Ortiz E., Cueto-Martín B., Sánchez-Gallardo I. (2017). Educación Deportiva versus Enseñanza Tradicional: Influencia sobre la regulación motivacional en alumnado de Bachillerato. Cuad. Psicol. Deporte.

[21] Gil-Arias A., Harvey S., Cárceles A., Práxedes A., Del Villar F. (2017). Impact of a hybrid TGfU-Sport Education unit on student motivation in physical education. PLoS ONE.

[22] Medina-Casaubón J., Burgueño R. (2017). Influencia de una temporada de educación deportiva sobre las estrategias motivacionales en alumnado de bachillerato: Una visión desde la teoría de la auto-determinación. Rev. Cienc. Deporte.

[23] Wallhead T.L., Garn A.C., Vidoni C. (2014). Effect of a sport education program on motivation for physical education and leisure-time physical activity. Res. Q. Exerc. Sport.

[24] Choi S.M., Sum K.W.R., Leung F.L.E., Wallhead T., Morgan K., Milton D., Ha S.C.A., Sit H.P.C. (2020). Effect of sport education on students’ perceived physical literacy, motivation, and physical activity levels in university required physical education: A cluster-randomized trial. High. Educ..

[25] Gil-Arias A., Harvey S., García-Herreros F., González-Víllora S., Práxedes A., Moreno A. (2020). Effect of a hybrid teaching games for understanding/sport education unit on elementary students’ self-determined motivation in physical education. Eur. Phys. Educ. Rev..

[26] Wallhead T.L., Garn A.C., Vidoni C. (2013). Sport Education and social goals in physical education: Relationships with enjoyment, relatedness, and leisure-time physical activity. Phys. Educ. Sport Pedagog..

[27] Knowles A., Wallhead T., Readdy T. (2018). Exploring the Synergy Between Sport Education and In-School Sport Participation. J. Teach. Phys. Educ..

[28] Perlman D., Goc Karp G. (2010). A self-determined perspective of the Sport Education Model. Phys. Educ. Sport Pedagog..

[29] Smither K., Xihe Z. (2011). High school students’ experiences in a Sport Education unit: The importance of team autonomy and problem-solving opportunities. Eur. Phys. Educ. Rev..

[30] Whitehead M., Durden-Myers E., Pot N. (2018). The Value of Fostering Physical Literacy. J. Teach. Phys. Educ..

[31] Pill S. (2008). A teacher’s perceptions of the sport education model as an alternative for upper primary school physical education. ACHPER Aust. Healthy Lifestyles J..

